# Correlates of male involvement in maternal and newborn health: a cross-sectional study of men in a peri-urban region of Myanmar

**DOI:** 10.1186/s12884-015-0561-9

**Published:** 2015-05-27

**Authors:** Frances Ampt, Myo Myo Mon, Kyu Kyu Than, May May Khin, Paul A. Agius, Christopher Morgan, Jessica Davis, Stanley Luchters

**Affiliations:** Burnet Institute, Melbourne, Australia; School of Public Health and Preventive Medicine, Monash University, Melbourne, Australia; Department of Medical Research (Lower Myanmar), Ministry of Health, Yangon, Myanmar; Faculty of Medicine, Dentistry and Health Sciences, University of Melbourne, Melbourne, Australia; Burnet Institute, Yangon, Myanmar; Judith Lumley Centre, Faculty of Health Sciences, La Trobe University, Melbourne, Australia; Melbourne School of Population and Global Health, University of Melbourne, Melbourne, Australia; International Centre for Reproductive Health, Department of Obstetrics and Gynaecology, Ghent University, Ghent, Belgium

**Keywords:** Male involvement, Maternal and newborn health, Maternal, newborn and child health, Women’s health, Gender and health, Myanmar, Burma, South East Asia, Sexual and reproductive health, Cross-sectional studies

## Abstract

**Background:**

Evidence suggests that increasing male involvement in maternal and newborn health (MNH) may improve MNH outcomes. However, male involvement is difficult to measure, and further research is necessary to understand the barriers and enablers for men to engage in MNH, and to define target groups for interventions. Using data from a peri-urban township in Myanmar, this study aimed to construct appropriate indicators of male involvement in MNH, and assess sociodemographic, knowledge and attitude correlates of involvement.

**Methods:**

A cross-sectional study of married men with one or more children aged up to one year was conducted in 2012. Structured questionnaires measured participants’ involvement in MNH, and their sociodemographic characteristics, knowledge and attitudes. An ordinal measure of male involvement was constructed describing the subject’s participation across five areas of MNH, giving a score of 1–4. Proportional-odds regression models were developed to determine correlates of male involvement.

**Results:**

A total of 210 men participated in the survey, of which 203 provided complete data. Most men reported involvement level scores of either 2 or 3 (64 %), with 13 % reporting the highest level (score of 4). Involvement in MNH was positively associated with wives’ level of education (AOR = 3.4; 95 % CI: 1.9-6.2; p < 0.001) and men’s level of knowledge of MNH (AOR = 1.2; 95 % CI: 1.1-1.3; p < 0.001), and negatively correlated with number of children (AOR = 0.78; 95 % CI: 0.63-0.95; p = 0.016).

**Conclusions:**

These findings can inform the design of programs aiming to increase male involvement, for example by targeting less educated couples and addressing their knowledge of MNH. The composite index proved a useful summary measure of involvement; however, it may have masked differential determinants of the summed indicators. There is a need for greater understanding of the influence of gender attitudes on male involvement in Myanmar and more robust indicators that capture these gender dynamics for use both in Myanmar and globally.

**Electronic supplementary material:**

The online version of this article (doi:10.1186/s12884-015-0561-9) contains supplementary material, which is available to authorized users.

## Background

Strategies to improve maternal, newborn and child health (MNCH) tend to focus on women. While education and empowerment of women are critically important, there is a limit to the gains that can be made when male partners are not considered [1–3].

In many societies, men are responsible for the decisions that directly impact their partners’ and children’s health, such as the use of contraceptives, access to health services, food quality and availability, and women’s workload [4]. Men may therefore play the role of ‘gatekeepers’ to health care [1, 5, 6], despite the fact that they often lack relevant knowledge [7]. Men who are poorly informed or disengaged from pregnancy and childbirth may present serious barriers to women’s ability to act in their own and their children’s interests.

Despite their frequent position as primary decision-maker, men tend to be excluded from health services and spaces in which they could learn more about family planning, pregnancy and childbirth. The exclusion can be sociocultural, in that pregnancy and childbirth is often considered ‘women’s business’, and there are economic drivers for men to work away from home [4]. It can also be programmatic; an exclusive focus on women in maternal health programs may result in health services that are inaccessible to men [8]. This exclusion may mean that men are less able to make informed decisions about reproductive and maternal health, and less willing to engage in such decision-making with their partners [4]. For example, the omission of men from family planning programs may have placed the burden of contraceptive decision-making onto women [9].

Men’s involvement has been tested in different contexts and found to be beneficial in a number of domains, including safer birth practices [10], family planning [11–15], HIV prevention [16, 17], maternal workload, birth preparedness and emergency obstetric access [18, 19], and partner communication and emotional support [4, 15, 18, 20]. In addition to the benefits for women and children, male involvement has potential benefits for men. These include improved quality of paternal and couple relationships, a more valued and constructive role for men, and increased access to, and familiarity with, the health system [21].

However, there are potential risks associated with male involvement programs if they are implemented in a way that is not sensitive to existing gender norms [1, 22]. They may reinforce gender stereotypes and isolate single women from services, and may even result in decreased service uptake by women, particularly in the case of HIV prevention [23].

To minimise these risks, it is important to ensure women have a central place in program design and evaluation [4, 24]. In addition, interventions in which a positive model of masculinity is promoted and men act as agents of change have been found to be particularly successful [2, 13, 14, 19].

### Defining male involvement

Male involvement has been variously defined, with two broad theoretical approaches emerging from the literature. The first considers male involvement to be a marker of gender equity as part of a social determinants of health framework [25]. Adopting more equitable gender roles such as joint decision-making within couples and shared control of household tasks or parenting is posited to lead to healthier behaviours and improved care-seeking. The second approach sees male involvement as more instrumental; the direct assistance provided by men to improve their partners’ and children’s health through the perinatal period. This approach is ‘gender-neutral’ [24] or ‘gender-blind’ [6] in that it considers men’s actions independent of their gendered roles. In fact, there is a risk that it may reinforce gender norms that disempower women [3, 26].

These approaches are two ways of conceptualising male involvement rather than categories of the different ways in which men can be involved. Evidently, practical activities such as helping with housework or attending childbirth may also challenge gender norms. The difference is that an instrumental approach sees the behaviour (such as attending birth) as an end in itself, whereas a gender equity approach examines its potential to combat gender inequities that contribute to poor health.

It is difficult to measure male involvement in a way that captures both the practical assistance provided by men to women, and the many ways that men can challenge prevailing gender norms. There are no established indicators for measuring involvement in the literature [8], and few authors are explicit about their own notions of involvement or their choice of indicators. Different indicators have been used to represent different types of involvement, including inter-spousal communication, attendance at antenatal care (ANC) and childbirth, and support provided during pregnancy. Each indicator used on its own cannot be said to constitute involvement [9], and some authors have combined multiple indicators into an index to capture a broader notion of involvement [3, 8, 21, 27].

### Male involvement in Myanmar

The limited literature available on the role of men in MNCH in Myanmar is consistent with that of other countries [28–30]. Despite high levels of female education and workforce participation, Myanmar is a patriarchal society in which men tend to dominate decisions that affect their family, but are restricted by social norms that consider MNCH to be a women-only domain. Available research suggests low levels of male involvement in reproductive health, including in relation to HIV and antenatal services [29], and a lack of open communication about sex between partners [30].

Several published studies have assessed the correlates or determinants of male involvement, however few of these use multivariable analysis to adjust for potential confounders, and we identified only one based in Myanmar [29]. This research is necessary to understand the factors that may influence some men to be more involved than others, and to define appropriate target groups for male involvement interventions.

Given the knowledge gap on the role of men in the region, the Burnet Institute (Melbourne and Myanmar offices), and the Department of Medical Research, Lower Myanmar (DMR), Ministry of Health, undertook a cross-sectional survey of men in a peri-urban setting in Myanmar, assessing their knowledge, attitudes and practices in relation to involvement in MNCH.

This analysis aimed to construct appropriate measures of male involvement in MNH, and develop a composite index based on these measures. Moreover, it assessed sociodemographic, knowledge and attitude correlates of male involvement in MNH.

## Methods

### Study design and participants

A cross-sectional design was employed. Married men with a child up to one year of age living in the study area, and available for interview, were eligible to participate. Between July and September 2012, participants were recruited from eleven clusters (local government areas known as ‘wards’). Each ward was defined as a geographically separate cluster.

### Study setting

The study area consisted of ten residential wards and one industrial ward (where many of the participants worked) in South Dagon Township, a satellite township two hours north of Yangon, the largest city in Myanmar. The estimated population of the study area is 100,000, with between 1,000 and 5,000 households per ward (locally sourced 2012 data; personal communication, Burnet Institute Myanmar, 2013). South Dagon has a population of approximately 269,460 across 32 wards [31]. Manual labour in local factories is the main form of employment. Basic maternal, neonatal and child health services are provided by local midwives responsible for each ward.

### Study procedures

Midwives selected eligible men from immunisation records, cross-referencing local birth and death registries, aiming to purposively recruit 20 men per ward. The midwives contacted eligible men via their wives, informed them about the survey and invited them to participate on a specified date. The midwife responsible for the industrial ward recruited ten eligible men from a local factory, with the factory owner’s permission.

A structured questionnaire was piloted on 20 men before being administered to the participants. The final questionnaire is available with this manuscript (see Additional file 1). Men were interviewed in Burmese by trained research assistants from DMR. The men were interviewed at private locations including at home, workplace and temple, on both weekdays and weekends. None of the men approached by the fieldwork team refused to participate. Each interview took between 30 and 45 min and was held in private with only the participant and one interviewer present. Participants were given a small gift for their time spent on the study.

### Ethical considerations

Written information was provided in Burmese and written consent obtained. A verbal explanation was provided for illiterate participants, who consented by marking the form with a thumbprint. Consent forms were stored in a secure location at DMR. Ethical approval for the study was obtained from the Proposal and Ethical Review Committee of DMR (Lower Myanmar), Ministry of Health Myanmar (approval number 36/ethics 2012). Additional statistical analysis was exempted from ethical review by the Monash University Human Research Ethics Committee (CF13/1149 – 2013000574), given the lack of additional risk posed to participants.

### Study measures

#### Outcome

The main outcome measure was a composite of five indicators of male involvement based on responses to questions about participation in specific areas of MNH: accompanying female partner to ANC at least once during the most recent pregnancy; father’s presence at the most recent childbirth; discussion of the pregnancy and birth with a health care provider; shared decision-making with their female partner regarding the antenatal and delivery care provider; and shared contraceptive decision-making. The rationale for selecting these indicators, and the potential limitations of using each alone as a measure of male involvement, are given in Table 1.

**Table 1:** Indicators of male involvement in the composite index

Indicator of male involvement	Rationale for inclusion	Limitations	Binary variable	Score in index
Accompanying female partner to ANC at least once during the most recent pregnancy	Most common definition of involvement used in the literature	Anecdotally, husband would rarely join wife in the consultation in Myanmar; instead may wait in the waiting room or outside*	1: Participant sometimes, mostly or always accompanied their wife to ANC	1
		Broad meaning of ‘accompany’; non-specific measure of involvement	0: Never accompanied	
Father’s presence at the most recent childbirth	Common indicator in the literature	Broad meaning of ‘presence’; non-specific measure of involvement	1: Present	1
			0: Not present	
	Presence in the room during labour is culturally inappropriate in Myanmar,* but presence nearby allows husband to support his wife and newborn in other ways			
Discussion of partner’s most recent pregnancy and birth with a health care provider	May indicate a greater depth of involvement and a greater readiness to assist	May indicate a male-dominant approach to pregnancy care	1: Discussion between participant and provider	1
		Not seen elsewhere in the literature	0: No discussion (or participant doesn’t recall, n = 1)	
Shared decision-making regarding antenatal and delivery care provider with their partner during the last pregnancy	Reflective of inter-spousal communication, which is an important component of involvement and impacts on MNH outcomes	Subject to recall and social desirability biases	1: Both participant and wife made the decision	1
		Doesn’t describe the extent or depth of communication within couples	0: Participant, wife, older family members or others made the decision	
Shared contraceptive decision-making with partner	Inter-spousal communication in the context of contraceptive use is well examined in the literature and associated with positive family planning outcomes			1

*Personal communication, Burnet Institute Myanmar, 2013

The index was developed to score involvement with improved content validity, capturing a broader notion of male involvement. Binary responses from these five questions indicating that the respondent participated in a particular aspect of MNH were summed to give a composite involvement index score, with higher index scores indicating more involvement. Given few respondents indicated either no participation (n = 2) or a single act of participation (n = 13), the lowest level in the regression model was grouped to include men reporting up to two acts of involvement, yielding a four level ordinal measure of MNH involvement.

#### Independent variables

Male survey respondents reported their own and their wives’ sociodemographic characteristics. Socioeconomic status was measured by household income and education levels of both partners. Income was categorised as greater or less than 100,000 kyats (approximately USD100), based on a 2003 estimate of average monthly household income for married labourers in Yangon’s informal sector [32], a demographic group similar to the survey population.

Men’s knowledge of MNH was measured using a single unit, constructed from a weighted composite scale comprising 39 items across five domains of knowledge, with scale units representing a correct answer to a knowledge question. Scores on the scale could range from 0 to 39, and had acceptable reliability (Cronbach’s α = 0.69).

A dichotomous measure of attitude towards male involvement in MNH was derived from responses to six normative statements (Table 2). These statements were selected from the survey based on their face validity – they reflect men’s expected roles in terms of their direct assistance, relevant knowledge and communication with their spouses. Responses to the six statements exhibited reasonable internal consistency (Cronbach’s α = 0.59). Men who gave responses in favour of male involvement for all six statements were considered to have a favourable attitude to male involvement.

**Table 2:** Attitude statements reflecting the role of men

Domain of expectations for the role of men	Attitude statement
Direct assistance with pregnancy and child rearing	Husbands do not need to help with child care
Knowledge to facilitate involvement	Husbands do not need to know the danger signs during pregnancy and childbirth
	Husbands do not need to have contraceptive knowledge
Inter-spousal communication and shared decision-making	Contraceptive decision making depends only on wife
	Men should tell their wives if they have contracted an STI
	Men should not disclose to their wives they have contracted HIV infection

### Analysis and data management

The questionnaire was translated into English prior to analysis. Data were double-entered into EpiData version 16 (EpiData Data Entry, Data Management and basic Statistical Analysis System. Odense Denmark: EpiData Association) and all analyses undertaken using STATA version 13 (StataCorp 2013. Stata Statistical Software: Release 13. College Station, TX: StataCorp LP). Proportional-odds regression models were used to explore correlates of male involvement in MNH, given the ordinal nature of male involvement. Brant tests [33] indicated that the final specified model did not violate the proportional-odds assumption (*χ*^2^(20) = 27.0; p = 0.135). Independent sociodemographic, attitude and knowledge factors were included in the regression models, including factors that were considered of interest a priori; namely age and level of education (of men and their wives), number of children, and income.

Model-based predicted marginal probabilities were compared across exposure groups for each level of male involvement in MNH, holding other model covariates at their mean. In all estimation, variance estimates (Huber-White sandwich variance estimator [34]) took account of ward clustering and the lack of independence in observations.

## Results

### Demographic characteristics of respondents

A total of 210 men participated in the survey, with a mean of 20 participants per ward (standard deviation (SD) = 3.0). Of the 210 respondents, seven were excluded from the analysis due to missing outcome data. Analysis of the demographic characteristics of those excluded did not suggest any bias (data not shown).

Male respondents were older than the reported age of their wives, with median ages of 32 and 30 years respectively (paired *t*-test t = 8.8; p < 0.001; Table 3). Level of education was similarly distributed among men and their wives, and illiteracy was very low (2 % for men and 3 % for their wives). Only two men (1 %) were unemployed, with the majority of men (54 %) working in insecure employment such as odd jobs or as casual staff on daily wages. The majority of men reported their wives to be unemployed (68 %). The most common form of employment for women was working in their own business (13 %). Nearly all respondents identified as Buddhist (96 %). Respondents most commonly had one child (49 %), and only 11 % had more than three children.

### Male involvement in MNH

A large majority of men accompanied their wives to ANC (82 %) and for delivery (87 %); the majority also shared health-related decisions with their wives (61 % and 69 % for decisions regarding health care provider and contraception respectively). In contrast, only one quarter (27 %) discussed their wives’ pregnancy with a health care provider (Table 3). Overall, 26 men (13 %) participated in all these acts of involvement, resulting in the highest index score of 4. Twenty-three percent reported the lowest score of 1 (representing up to two acts of involvement). The median index score was 2 (interquartile range (IQR) = 2-3), with the same number of men reporting scores of 2 and 3 (32 % respectively).

**Table 3:** Sociodemographic, knowledge and attitude characteristics of survey respondents, and levels of male involvement in MNH by indicator: N = 203

Sociodemographic characteristics	
Respondent’s age in years: median (IQR)	32 (27–38)
Wife’s age in years: median (IQR)	30 (24–34)
Education: n (%)	
Illiterate	4 (2 %)
Primary/basic literacy	47 (23 %)
Middle school	83 (41 %)
High school/university	69 (34 %)
Wife’s education: n (%)	
Illiterate	6 (3 %)
Primary/basic literacy	54 (27 %)
Middle school	68 (34 %)
High school/university	75 (37 %)
Employment: n (%)	
Unemployed	2 (1 %)
Odd jobs or daily wager	109 (54 %)
Private or government employee or own business	79 (39 %)
Other	13 (7 %)
Wife’s employment: n (%)	
Unemployed	139 (68 %)
Odd jobs or daily wager	18 (9 %)
Private or government employee or own business	36 (18 %)
Other	10 (5 %)
Number of children: median (IQR)	2 (1–3)
Household income (USD/month)*: n (%)	
≤100	95 (47 %)
>100	108 (53 %)
Religion: n (%)	
Buddhist	194 (96 %)
Muslim	7 (3 %)
Other	2 (1 %)
Knowledge of MNH and attitudes towards male involvement	
Knowledge score: median (IQR)	8 (6–10)
Favourable attitude to male role in MNH: n (%)	78 (38 %)
Indicators of male involvement: n (%)	
Accompanied wife to ANC	166 (82 %)
Present at the place of childbirth	177 (87 %)
Discussed pregnancy with a health provider	55 (27 %)
Shared decision regarding health care provider	123 (61 %)
Shared decision regarding contraception	141 (69 %)

* Equivalent to 100,000 Myanmar kyats

### Factors associated with male involvement in MNH

In terms of unadjusted associations, men with high school or university education (odds ratio (OR) = 3.3; 95 % confidence interval (95 % CI) = 2.0-5.3), highly educated wives (OR = 4.4; 95 % CI = 2.8-6.8), greater sexual and reproductive health knowledge scores (OR = 1.2; 95 % CI = 1.1-1.3), and favourable attitudes towards male involvement (OR = 1.9; 95 % CI = 1.2-3.2), were more likely to demonstrate higher levels of involvement in MNH (Table 4). Wife’s education and men’s knowledge remained significantly associated with involvement after adjustment for other factors. Men whose wives had finished at least high school were more than three times as likely to have higher levels of involvement in MNH than those whose wives only had primary level education (adjusted odds ratio (AOR) = 3.4; 95 % CI = 1.9–6.2; p < 0.001); these men were also more likely than those whose wives had ‘middle’ school education to have higher involvement (Wald *χ*^2^(1) = 8.46, p = 0.004). In terms of knowledge of MNH, men’s odds of involvement in MNH increased by an average of 20 % for each correct answer to the set of MNH knowledge questions (AOR = 1.2; 95 % CI = 1.1–1.3; p < 0.001). Number of children also exhibited a significant independent association in the multivariable model, with the odds of being involved in MNH decreasing by 22 % for every additional child (AOR = 0.78; 95 % CI = 0.63–0.95; p = 0.016).

**Table 4:** Factors associated with male involvement in MNH: Proportional-odds regression models showing unadjusted and adjusted odds ratios, 95 % confidence intervals and probability values (n=203)*

Independent variable	Unadjusted OR	95 % CI†	p-value	Adjusted OR	95 % CI†	p-value
Sociodemographic factors						
Respondent’s age	0.99	0.97 – 1.02	0.616	0.99	0.96 - 1.02	0.481
Respondent’s wife’s age	1.00	0.97 – 1.03	0.893	1.02	0.98 - 1.06	0.348
Education						
Up to primary	1.00			1.00		
Middle school	1.33	0.80 – 2.21	0.240	0.72	0.42 – 1.24	0.237
High school/ university	3.26	2.02 – 5.27	<0.001	1.10	0.57 - 2.12	0.780
Wife’s education						
Up to primary	1.00			1.00		
Middle school	1.53	0.86 – 2.73	0.130	1.43	0.91 – 2.25	0.116
High school/university	4.36	2.80 – 6.80	<0.001	3.39	1.90 – 6.15	<0.001
Number of children	0.81	0.65- 1.01	0.057	0.78	0.63 – 0.95	0.016
Household income (USD/month)						
≤100	1.00			1.00		
>100	1.20	0.64 – 2.24	0.527	1.20	0.62 – 2.31	0.591
Knowledge of MNH						
Knowledge score	1.20	1.11 – 1.29	<0.001	1.18	1.10 – 1.27	<0.001
Overall attitude to male role in MNH (attitude scale)						
Unfavourable	1.00			1.00		
Favourable	1.92	1.16 – 3.17	0.016	1.44	0.93 – 2.23	0.105

* Multivariable model cut-points: k_1_ = 0.47, k_2_ = 2.13, k_3_ = 4.13

† Sandwich variance estimation used to provide appropriate standard errors for village clustering

**Fig. 1:**
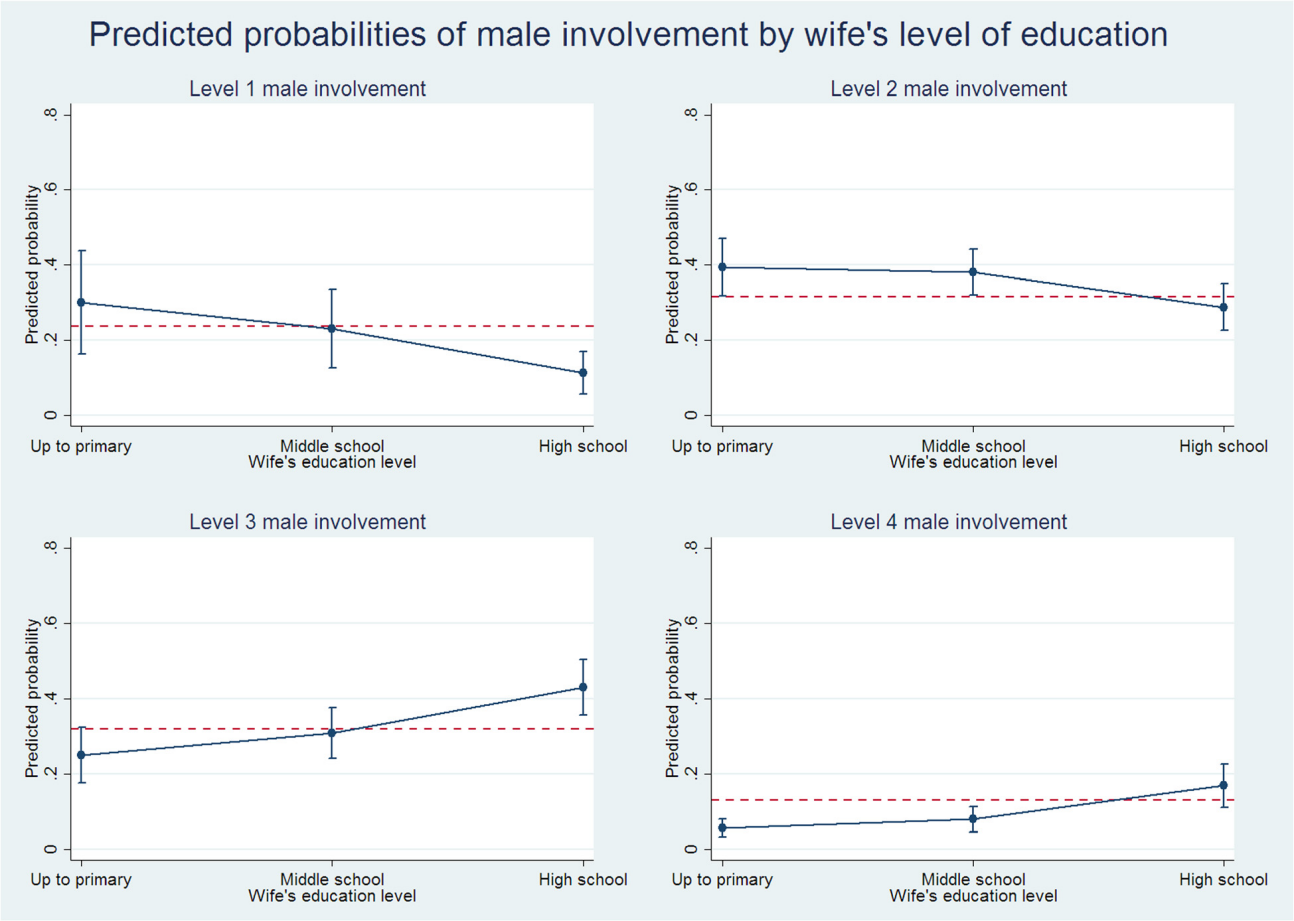
Plots of predicted marginal probabilities of each of the four levels of male involvement by wife’s level of education. The red dashed line represents the base level probability of male involvement at the specified level. Whiskers represent 95 % confidence intervals around each probability estimate

**Fig. 2:**
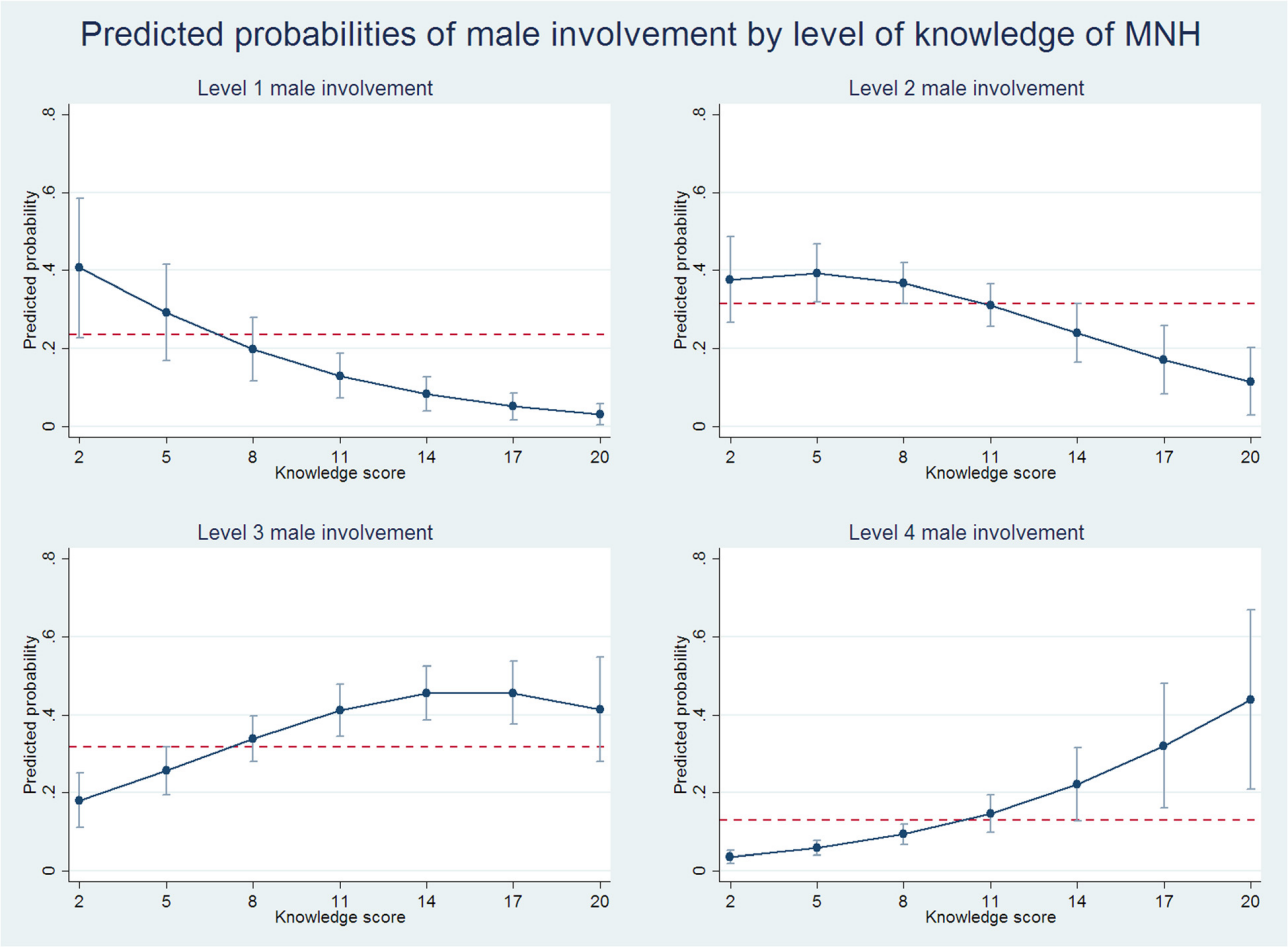
Plots of predicted marginal probabilities of each of the four levels of male involvement by level of knowledge of MNH

**Fig. 3:**
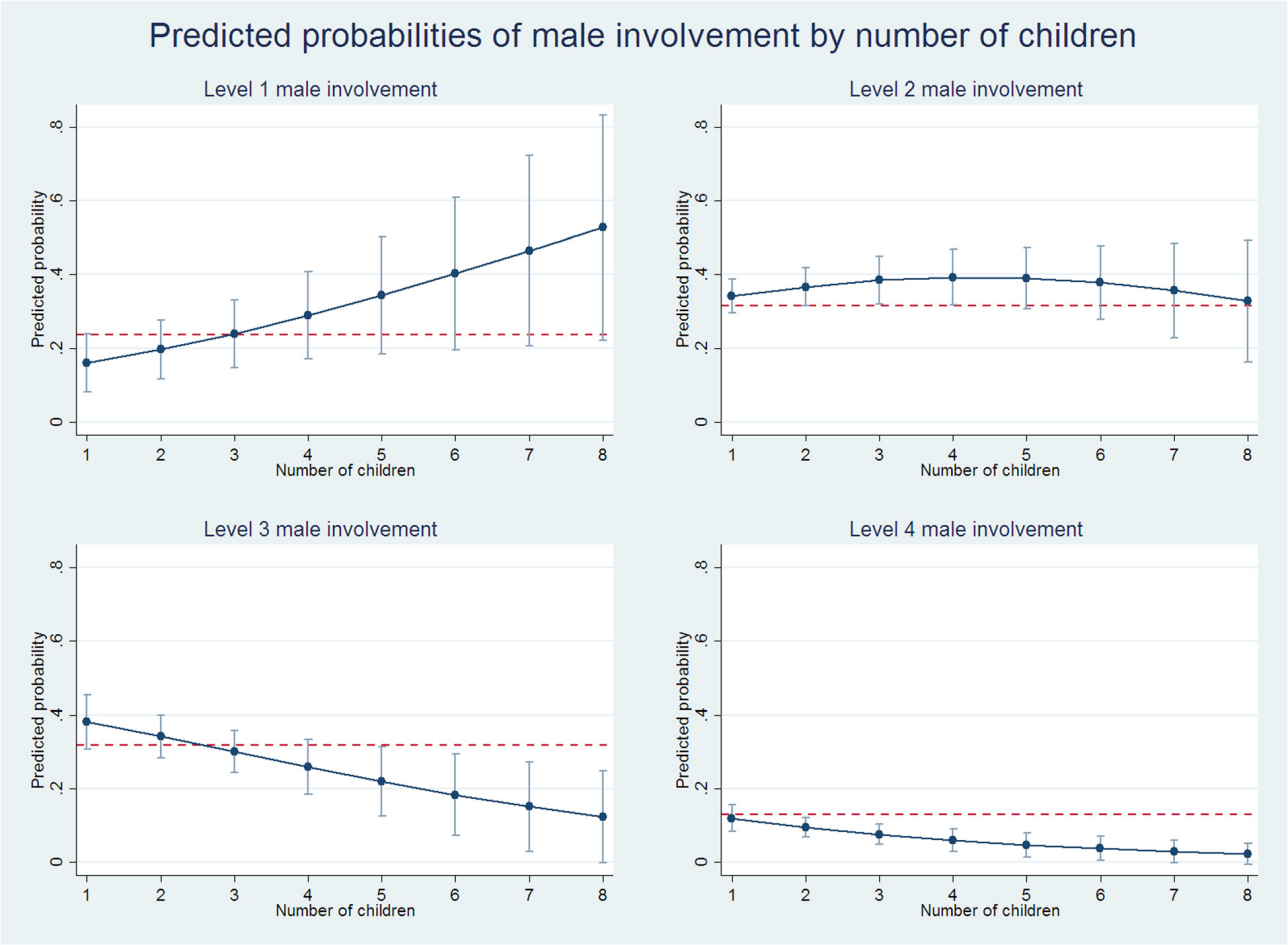
Plots of predicted marginal probabilities of each of the four levels of male involvement by number of children

Marginal probabilities from the proportional-odds model further describe the nature of relationships between male involvement in MNH and key factors. Figs. 1, 2 and 3 show plots of model-based marginal probabilities, by wife’s education, knowledge of MNH and number of children, respectively. For instance, compared to those whose wives had primary education only, men whose wives had achieved at least high school education were less likely to indicate involvement in MNH at lower levels (level 1: 11.3 % vs. 30.1 %; level 2: 28.8 % vs. 39.3 %), but exhibited greater probability of higher involvement in MNH (level 3: 43.1 % vs. 25.0 %; level 4: 16.8 % vs. 5.6 %) (Fig. 1). Comparing the plots across outcome levels shows the greater overall probability of male involvement at levels 2 and 3 (see the reference y-line in plots), and this reflects the higher observed prevalence at these levels.

## Discussion

### Correlates of male involvement in MNH

Among the study population in South Dagon, women’s level of education is independently correlated with their husbands’ involvement in MNH. Women’s education is recognised as one of the key predictors of women’s and children’s health, and this analysis suggests that educating women also facilitates their partners’ engagement in their health and that of their children. Other studies have found an association between women’s education and male involvement, particularly in relation to shared decision-making [3, 35–37]. However, this positive association is not universal; for example, Olayemi et al. found that while the husbands of educated women were more likely to assist with household chores, they were less likely to attend ANC [38]. It may be that women who are better educated have a more assertive role in the household and that such a role is not necessarily conducive to all aspects of male involvement. For example, an analysis of national data from sub-Saharan Africa found a mixed relationship between measures of women’s empowerment and their male partner accompanying them to ANC, with different countries showing positive, negative and non-significant associations [39].

We also found, as have others [40], that men with greater levels of knowledge about sexual and reproductive health are more likely to be involved in their wives’ pregnancies and newborn care. This may be a result of men learning through their involvement, for example gaining knowledge of high-risk pregnancies after accompanying their wife to ANC. Conversely, men may become more involved because greater knowledge makes them aware of the potential dangers of pregnancy and childbirth and the importance of care-seeking; in this case, interventions aiming to improve men’s knowledge of sexual and reproductive health may have a positive impact on their degree of involvement.

Our finding that families with more children had lower male involvement, suggests that family size may influence men’s participation in maternal and newborn health. While this is not a common finding, Tweheyo et al. found that the intention to have more children was associated with lower male attendance at ANC [40]. Achieving positive male involvement in care during successive pregnancies may require targeted attention.

While men’s attitude and level of education were associated with level of involvement in bivariate analyses, these associations did not hold when other factors were taken into account in multivariable analyses. However, it is feasible that such factors are implicated in the complex pathways between knowledge and involvement. Our results may have differed had we used a more sensitive and reliable measure of gender-equity attitudes rather than the general attitude statements used in this study, which was limited by time and resources. For example, the Gender-Equitable Men scale has been validated for use in different cultural contexts [41], and similar indices have been found to correlate with aspects of male involvement [35, 42].

Other interventional research shows that gender-transformative male involvement programs, which challenge inequitable gender roles and structures, are more likely to be successful and sustainable than those which take a ‘gender-sensitive’ or ‘gender-neutral’ approach [24]. For example, gender-transformative interventions like the Malawi male motivator project [14] and family planning education in El Salvador [13] have been successful in improving inter-spousal communication and contraceptive uptake. This evidence, when paired with our study’s findings, suggest that educational interventions to improve male involvement in Myanmar should target poorly educated couples, both first pregnancies and couples with larger families, as well as exploring and addressing underlying attitudes and behaviours relating to gender roles and spousal communication. In addition, while we only studied couples with children, interventions that use a life-course approach to reach young boys and men, before they establish relationships and have children, may be more successful in changing attitudes and behaviours. However, such programs are rare [43].

### Measuring male involvement in MNH

Defining and measuring involvement is a methodological challenge. In this study, the development of a composite index was a feasible method of encapsulating multiple aspects of involvement and addressing the limitations associated with more narrow indicators. Several other studies have used composite indices to represent male involvement. While the indicators chosen vary and no indices have been well validated, other studies do provide a point of reference for our methods. As in Carter and Speizer [21] and Iliyasu et al. [27], our study includes men’s attendance at ANC and birth as indicators of practical involvement. It was also considered important to capture aspects of inter-spousal communication, as have Mullany et al. [3] and Byamugisha et al. [8]. This was characterised in our survey as ‘shared decision-making’, a proxy for communication used in the literature [35] and associated with other types of involvement [3].

It is possible that combining the five indicators in an index masked their differential characteristics. This loss of granularity is likely to be a limitation of similar composite indices in the literature [3, 44]. Perhaps it is an oversimplification to consider ‘male involvement’ as a singular construct that can be consistently defined. It may be more helpful to generate a consensus framework describing distinct domains of male involvement (such as practical participation in services, or inter-spousal communication) and measure each individually. These domains could be derived from the theoretical models of involvement, bringing greater meaning to the conceptualisation of male involvement and its determinants.

### Limitations

There are a number of methodological issues that limit the interpretation and generalisability of this study. Given that men were purposively selected from wards, the extent to which the results from this study are generalisable to the population of men in the southern Dagon region is limited. Furthermore the cross-sectional design precludes any inferences about the direction of relationships between male involvement and its correlates.

There are some potential sources of selection bias. First, selection from immunisation records recruits families already connected to health services. However, this is unlikely to significantly misrepresent the wider population as immunisation rates in South Dagon are high [31]. Secondly, convenience sampling by midwives may have selected men who were better connected with health services, more able and willing to engage, and who had more gender-equitable attitudes than the wider population. Finally, data collection was conducted on set days and eligible men could participate if they were at their home on the relevant day, so it is possible *under*employed men could have been overrepresented, though rates of *un*employment in the sample were very low.

Social desirability bias may have influenced responses, given the interviewers were from a government department and in a position of authority. This is particularly relevant to attitude questions. However, the dichotomous attitude measure was designed to better discriminate between men who were genuinely in favour of involvement (those who answered favourably to *all six* questions) and those who had answered some questions in this way to meet perceived expectations. There may also have been some recall bias, particularly in the measures of inter-spousal communication, as recollection of discussions during pregnancy could have been coloured by subsequent events.

Despite the limitations outlined, the methods adopted in this study had a number of advantages. The sampling process made good use of relationships with local administrators and health workers, and of existing connections within the community. The survey was developed and administered by local staff with a good understanding of the cultural context. Finally, our analytical approach to modelling involvement (proportional-odds regression), given its assumptions, avoids the more arbitrary definition of levels of involvement that would have applied had we adopted a conventional binary approach to measurement and estimation.

## Conclusions

Considering the lack of existing research on male involvement in MNH in the region [19, 29], this analysis represents a valuable snapshot of the correlates of male involvement in a peri-urban population in Myanmar. It provides a useful base for further research, particularly into links between different types of involvement and MNH outcomes, and qualitative research into why and how men in this cultural context become involved and the implications for their relationships and communities. The associations between higher levels of male involvement and knowledge, female partner education and number of children provide a useful reference for targeting male involvement interventions in the future.

## Additional file

Additional file 1:
**Study questionnaire.**
