# Supplementary material for: Correlates of male involvement in maternal and newborn health: a cross-sectional study of men in a peri-urban region of Myanmar
Source: BMC Pregnancy Childbirth. 2015 May 27;15:122. doi: 10.1186/s12884-015-0561-9 (PMC4445797; doi:10.1186/s12884-015-0561-9)
Supplement: Additional file 1: — Study questionnaire. [file 12884_2015_561_MOESM1_ESM.pdf]

Additional file 1: Study questionnaire

Section I Background characteristics

1.1	Respondent ID		|__|__|__|
1.2	Ward		|___|___|
1.3	Respondent's age (completed year)	_________________ Yrs	|___|___|
1.4	Age of wife (completed year)	_________________ Yrs	|___|___|
1.5	Religion of the respondent	(1) Buddhist<br>(2) Christian<br>(3) Muslim<br>(4) Hindu<br>(5) Others (specify) _________	|___|
1.6	Education level of the respondent	(1) Illiterate<br>(2) Read and write<br>(3) Primary school<br>(4) Middle school<br>(5) High school<br>(6) University/Graduate<br>(7) Post graduate	|___|
1.7	Education level of wife	(1) Illiterate<br>(2) Read and write<br>(3) Primary school passed<br>(4) Middle school passed<br>(5) High school passed<br>(6) University/Graduate<br>(7) Post graduate	|___|

1.8	Occupation of the respondent	(1) Unemployed<br>(2) Odd job (Kya-ban)<br>(3) Daily wager<br>(4) Private employee<br>(5) Government employee<br>(6) Own business<br>(7) Others (specify) _________	|___|
1.9	Occupation of wife	(1) Unemployed<br>(2) Odd job (Kya-ban)<br>(3) Daily wager<br>(4) Private employee<br>(5) Government employee<br>(6) Own business<br>(7) Others (specify) _________	|___|
1.10	Number of children	_____________________	|___|___|
1.11	Estimated monthly family income (Ks)	(1) <50,000<br>(2) 50,000 – 100,000<br>(3) >100,000 – 200,000<br>(4) >200,000 – 500,000<br>(5) >500,000	|___|

Section II Awareness, knowledge, perception and practice of men regarding safe motherhood

**II A. Knowledge**			
2.1	Does a pregnant woman need antenatal care?	(1) Yes<br>(2) No (go to 2.3)<br>(3) Don't know (go to 2.3)	|___|
2.2	From whom a pregnant woman should receive antenatal care?	(1) Obstetrics and gynecologist<br>(2) Other medical doctor	|___|

		(3) HA/LHV<br>(4) Midwife<br>(5) Auxiliary midwife<br>(6) Traditional birth attendant<br>(7) Others --------------------	
2.3	How many times should a pregnant woman receive antenatal care? (at least)	-----------------------------	|___|___|
2.4	Which antenatal care services are provided to pregnant women during ANC visit? (Multiple response)	(1) Tetanus injection<br>(2) Iron tablets<br>(3) Abdominal examination<br>(4) Blood pressure measurement<br>(5) Deworming<br>(6) Others ---------------------	|___| |___| |___| |___| |___| |___|
2.5	Do you know the danger signs during pregnancy?	(1) Yes<br>(2) No (go to 2.7)	|___|
2.6	What are the danger signs during pregnancy? (Multiple response)	(1) Severe vomiting<br>(2) Bleeding per vagina<br>(3) Hypertension<br>(4) Swelling of face and feet<br>(5) High fever<br>(6) Eclampsia<br>(7) Fits<br>(8) Others --------------------------	|___| |___| |___| |___| |___| |___| |___| |___|
2.7	Do you know types of pregnant mothers who must deliver at the hospital?	(1) Yes<br>(2) No (go to 2.9)	|___|
2.8	What are these?<br>(Multiple response)	(1) First pregnancy<br>(2) Multipara (> children)<br>(3) Short stature (<4 Ft 10 In)	|___| |___| |___|

		(4) Multiple pregnancy<br>(5) Maternal age under 18<br>(6) Maternal age over 35<br>(7) Malposition/size of fetus<br>(8) Hypertension<br>(9) Others --------------------------	|___| |___| |___| |___| |___| |___|
2.9	Do you know the danger signs during delivery?	(1) Yes<br>(2) No (go to 2.11)	|___|
2.10	What are the danger signs during delivery? (Multiple response)	(1) Excessive bleeding<br>(2) Prolonged labor (>24 hours)<br>(3) Dirty or meconium stained liquor<br>(4) Maternal distress<br>(5) Fits<br>(6) Unconsciousness<br>(7) Retained placenta<br>(8) Others -------------------------	|___| |___| |___| |___| |___| |___| |___| |___|
2.11	Do you know the danger signs during postnatal period?	(1) Yes<br>(2) No (go to 2.13)	|___|
2.12	What are the danger signs during postnatal period? (Multiple response)	(1) Post partum hemorrhage<br>(2) High fever<br>(3) Puerperial psychosis<br>(4) Others -------------------------	|___| |___| |___| |___|
2.13	Do you know the danger signs of newborn baby?	(1) Yes<br>(2) No (go to 2.15)	|___|
2.14	What are the danger signs of newborn baby which need to seek health care? (Multiple response)	(1) Poor breast feeding<br>(2) Neonatal jaundice<br>(3) Umbilical sepsis<br>(4) Eye infection	|___| |___| |___| |___|

		(5) Others -------------------------	|___|
2.15	Do you know the vaccine preventable childhood diseases?	(1) Yes<br>(2) No (go to 2.17)	|___|
2.16	If yes, what are these diseases?<br>(Multiple responses)	(1) TB<br>(2) Diptheria<br>(3) Whooping cough<br>(4) Tetanus<br>(5) Polio<br>(6) Measles<br>(7) Hepatitis B	|___| |___| |___| |___| |___| |___| |___|
**II B. Perception**			
2.17	Husbands should participate in receiving antenatal care of his wife.	(1) Agree<br>(2) Not sure<br>(3) Disagree	|___|
2.18	Husbands are responsible for financial support during pregnancy and childbirth.	(1) Agree<br>(2) Not sure<br>(3) Disagree	|___|
2.19	Husbands do not need to know the danger signs during pregnancy and childbirth.	(1) Agree<br>(2) Not sure<br>(3) Disagree	|___|
2.20	Husbands should arrange to receive antenatal care.	(1) Agree<br>(2) Not sure<br>(3) Disagree	|___|
2.21	Husbands do not need to help in child care.	(1) Agree<br>(2) Not sure<br>(3) Disagree	|___|
2.22	Husbands are mainly responsible for correct decision making in emergency situation during pregnancy and delivery.	(1) Agree<br>(2) Not sure	|___|

		(1) Disagree	
**II C. Practice**			
2.23	Did your wife receive antenatal care for last pregnancy?	(1) Yes<br>(2) No (go to 2.27)<br>(3) Don't know (go to 2.27)	|___|
2.24	Who was ANC provider for last pregnancy?	(1) Obstetrics & Gynaecologist<br>(2) Medical doctor<br>(3) LHV<br>(4) MW<br>(5) AMW<br>(6) TBA<br>(7) Others ------------------------	|___|
2.25	Did you accompany your wife in receiving ANC for last pregnancy?	(1) Yes, always<br>(2) Most of the time<br>(3) Sometimes<br>(4) Never	|___|
2.26	If never accompany, why?	(1) Not free, need to work<br>(2) Need to take care of other children<br>(3) Think it's not necessary<br>(4) Since the place is only for women<br>(5) Others -------------------------	|___|
2.27	Did you accompany your wife for the delivery (or) present at the place?	(1) Yes<br>(2) No	|___|
2.28	Do you have experience of discussion with health care provider about your wife's last pregnancy and childbirth?	(1) Yes<br>(2) No<br>(3) Don't remember	|___|
2.29	Did you discuss with your wife regarding the choice of health care provider for the last birth?	(1) Yes<br>(2) No	|___|

		(1) Don't remember	
2.30	If yes, which type of provider did you recommend?	(1) Obstetrics & Gynaecologist<br>(2) Medical doctor<br>(3) LHV<br>(4) MW<br>(5) AMW<br>(6) TBA<br>(7) Others -------------------------	|___|
2.31	Who made decision for the choice of provider regarding antenatal and delivery care?	(1) Myself<br>(2) My wife<br>(3) Both of us<br>(4) Elder family members<br>(5) Others -------------------------	|___|
2.32	Where did your wife deliver last baby?	(1) Home<br>(2) RHC/sub-RHC<br>(3) Maternity home<br>(4) Township hospital<br>(5) Private hospital<br>(6) Tertiary hospital<br>(7) Others --------------------------	|___|
2.33	Who was the health care provider (birth attendant) for your wife's last birth? (Choose only one main provider)	(1) Obstetrics & Gynaecologist<br>(2) Medical doctor<br>(3) LHV<br>(4) MW<br>(5) AMW<br>(6) TBA<br>(7) Others -------------------------	|___|
2.34	Did your child (any children) need to seek health care for last six months?	(1) Yes	|___|

		(2) No (go to 3.1)<br>(3) Don't know (go to 3.1)	
2.35	If yes, who was the health care provider?	(1) Pediatrician<br>(2) Other medical doctor<br>(3) Nurse/MW<br>(4) TBA<br>(5) Others ---------------------------	|___|
2.36	Did you accompany in seeking health care for a child?	(1) Yes<br>(2) No	|___|
2.37	Who is the main decision maker to seek care for a child?	(1) Myself<br>(2) My wife<br>(3) Both of us<br>(4) Elder family members<br>(5) Others -------------------------	|___|

Section III Awareness, knowledge, perception and practice of men regarding contraception

**III A. Knowledge regarding contraception**			
3.1	Do you know contraceptive methods?	(1) Yes<br>(2) No (go to 3.4)	|___|
3.2	Contraceptive methods include:<br>(Multiple response)	(1) Oral daily pills<br>(2) One month injection<br>(3) Three month injection (Depo)<br>(4) Emergency pill<br>(5) Condom<br>(6) Intrauterine device<br>(7) Female sterilization<br>(8) Male sterilization<br>(9) Implant	|___| |___| |___| |___| |___| |___| |___| |___| |___|

3.3	Advantages of condom include:	(1) Prevention of pregnancy<br>(2) Prevention of HIV transmission<br>(3) Prevention of STI<br>(4) Others ---------------------------	|___| |___| |___| |___|
**III B. Perception regarding contraception**			
3.4	Husbands do not need to have contraceptive knowledge.	(1) Agree<br>(2) Not sure<br>(3) Disagree	|___|
3.5	Husbands should discuss with their wives about contraception.	(1) Agree<br>(2) Not sure<br>(3) Disagree	|___|
3.6	Decision for contraception should be made by both partners.	(1) Agree<br>(2) Not sure<br>(1) Disagree	|___|
3.7	Using condom may make one's partner uncomfortable.	(1) Agree<br>(2) Not sure<br>(3) Disagree	|___|
3.8	Men who have extramarital or paid sex should use condom.	(1) Agree<br>(2) Not sure<br>(3) Disagree	|___|
3.9	Contraceptive decision making depends only on wife.	(1) Agree<br>(2) Not sure<br>(3) Disagree	|___|
**III C. Practice regarding contraception**			
3.10	Are you/your wife using any contraceptive method now?	(1) Yes<br>(2) No (go to 3.12)<br>(3) Don't know (go to 3.12)	|___|
3.11	If yes, which method	-----------------------------	|___|

3.12	Who is the decision maker to use or not to use contraception?	(1) Myself<br>(2) My wife<br>(3) Both of us<br>(4) Elder family members<br>(5) Others ------------------------	|___|
3.13	Do you and your wife agree in contraceptive decision?	(1) Yes<br>(2) No	|___|

Section IV Awareness, knowledge, perception and practice of men regarding STI/HIV

**IV A. Knowledge regarding STI/HIV**			
4.1	Have you ever heard sexually transmitted infections?	(1) Yes<br>(2) No (go to 4.6)	|___|
4.2	If yes, signs and symptoms of STI include:	(1) Genital ulcer<br>(2) Burning urination<br>(3) Urethral discharge<br>(4) Inguinal lymph node enlargement<br>(5) Genital Herpes<br>(6) Genital warts<br>(7) Others --------------------------	|___| |___| |___| |___| |___| |___| |___|
4.3	Is there treatment for complete cure of STI?	(1) Yes<br>(2) No<br>(3) Don’t know	|___|
4.4	Is it necessary to treat a sexual partner at the same time if someone contracts STI?	(1) Yes<br>(2) No<br>(3) Don’t know	|___|
4.5	Can STI be transmitted from mother to fetus?	(1) Yes<br>(2) No<br>(3) Don’t know	|___|

4.6	Have you aware of HIV/AIDS?	(1) Yes<br>(2) No (go to 4.9)	|___|
4.7	What are the modes of transmission of HIV? (Multiple response)	(1) Sexual contact<br>(2) Blood transfusion<br>(3) Mother to fetus<br>(4) Through contaminated sharp utensils (needles, blades, etc)<br>(5) Others --------------------------	|___| |___| |___| |___| |___|
4.8	Does a pregnant woman need HIV testing?	(1) Yes<br>(2) No<br>(3) Don't know	|___|
4.9	How was HIV transmitted from mother to child?	(1) During pregnancy<br>(2) At the time of delivery<br>(3) During breast feeding<br>(4) Others ---------------------------	|___| |___| |___| |___|
**IV B. Perception regarding STI/HIV**			
4.10	Men should tell their wives if they have contracted STI.	(1) Agree<br>(2) Not sure<br>(3) Disagree	|___|
4.11	Men should not take his wife to treat STI together with him.	(1) Agree<br>(2) Not sure<br>(3) Disagree	|___|
4.12	Men should not disclose to their wives if they have contracted HIV infection.	(1) Agree<br>(2) Not sure<br>(3) Disagree	|___|
**IV C. Practice regarding HIV/STI**			
4.13	Do you have any experience of getting STI?	(1) Yes<br>(2) No (go to 4.15)	|___|

		(3) Don't know (go to 4.15)	
4.14	If yes, did you take any treatment?	(1) Yes<br>(2) No (go to 4.16)	|___|
4.15	From whom did you take treatment?	(1) Self medication<br>(2) From drug shop<br>(3) Traditional healer<br>(4) Quack<br>(5) Medical doctor<br>(6) STI specialist<br>(7) Others --------------------------	|___|
4.16	Have you ever received HIV testing?	(1) Yes<br>(2) No (go to 4.18)	|___|
4.17	Why did you take HIV test? (if more than one time, take the last reason)	(1) Test because wife is pregnant (PMCT)<br>(2) Advice from health care provider<br>(3) Because of high risk behavior<br>(4) Before operation<br>(5) Needs to test for a job<br>(6) To make/renew driving license<br>(7) Blood transfusion<br>(8) Others ------------------------	
4.18	Do you know the place for voluntary counseling and testing of HIV?	(1) Yes<br>(2) No (end)	|___|
4.19	What are these?	(1) ------------------<br>(2) ------------------	|___| |___|

Date –

Interviewer –
